# Study of autologous dendritic cell therapy targeting Mucin 1 as a treatment for the maintenance of ovarian cancer patients in remission

**DOI:** 10.1186/2051-1426-1-S1-P213

**Published:** 2013-11-07

**Authors:** J Goh, J Mason, J Chan, M Moradi, J Berek, B Beningno, L Mileshkin, F Recio, N Tchabo, E Rossi, P Eisenberg, P Rose, P Mitchell, J Young, M Matos, A Secord, M Davy, S Gargosky, H Gray

**Affiliations:** 1 Greenslopes, Brisbane, QLD, Australia; 2 ScrippsCC, La Jolla, CA, USA; 3 UCSF, San Francisco, CA, USA; 4 NYDT, New York, NY, USA; 5 Stanford, Palo Alto, CA, USA; 6 Northside, Atlanta, GA, USA; 7 PMCC, MELBOURNE, VIC, Australia; 8 CollResGrp, Boca Raton, FL, USA; 9 MorristownMC, Morristown, NJ, USA; 10 IndianaUni, Indianapolis, IN, USA; 11 MarinCC, Greenbrae, CA, USA; 12 ClevelandClin, Cleveland, OH, USA; 13 Austin, Melbourne, VIC, Australia; 14 MUSC, Charelston, NC, USA; 15 GoldCoast, Southport, QLD, Australia; 16 DukeUMC, Durham, NC, USA; 17 RAH, Adelaide, SA, Australia; 18 PrimaBioMed, Sydney, NSW, Australia; 19 UniWashington, Seattle, WA, USA

## 

CVac is an autologous cellular therapy targeted to elicit a T cell response to tumors that over-express mucin 1. CAN-003 is a randomized, open-label, Phase 2 trial evaluating the safety and efficacy of CVac given as a single agent to epithelial ovarian cancer (EOC) patients who are in complete remission (CR) following first or second-line chemotherapy. Patients were eligible if they had stage III or IV EOC and obtained a complete response to standard first or second line platinum/ taxane based chemotherapy. The first 7 patients received CVac to allow evaluation of manufacturing in the US and for safety evaluation. Patients were randomized to CVac therapy or standard of care (SOC). Patients in the active group were treated with up to 10 doses of CVac, 4 weekly for 7 doses, and 8 weekly for 3 doses. The trial is closed to enrolment and completes in 2013. 63 patients were enrolled into the trial; 42 were in 1st remission (age 55.7+/- 9.2 y) and 21 were in 2nd remission (age 56.8+/-9.0 y). 60 were Caucasian, 1 African American and 2 Asian. 36 treated patients and 27 OSC (data until Dec 12 2013). 10 SAEs were reported in total. 7 SAEs in CVac patients and 3 SAEs were reported in SOC; none were unexpected and only one (abdominal pain) was classified as possibly-related to CVac. Interim analysis has shown positive trends in PFS; as of 17 August 2012, the median PFS for days on study as 365 d for CVac, 421 d for non-randomized CVac, and 321 d for OSC. Updated PFS data will be presented at the meeting. Overall survival data is being collected and will be presented at the meeting. As expected, assessment of anti-mucin-1 responses have indicated no humoral response, however, interim immune monitoring results show a T cell response that is mucin 1-specific after 3 CVac doses. No mucin 1 response was observed in normal healthy volunteers nor untreated patients. The complete T-cell responses analysis throughout the course of CVac treatment will be presented at the meeting. In conclusion, study data show that immunotherapy with CVac is well tolerated. Immunological outcomes are consistent with the mechanism of action and the interim trends are encouraging for PFS.

